# Implementation research on osteoarthritis in Asia: a systematic review

**DOI:** 10.3389/fpubh.2026.1693849

**Published:** 2026-02-10

**Authors:** Ansuman Panigrahi, Purnashashi Behera, Swati Sambita Mohanty, Priyanka Sahu, Rutuparna Sibani Dandsena, Sanghamitra Pati

**Affiliations:** ICMR-Regional Medical Research Centre, Bhubaneswar, Odisha, India

**Keywords:** Asia, implementation research, osteoarthritis, StaRI checklist, systematic review

## Abstract

**Background:**

Osteoarthritis is a prevalent musculoskeletal disorder significantly impacting quality of life, especially in ageing populations. While effective interventions exist, their implementation in Asian healthcare settings is often lacking. This study aims to assess and collate existing evidence on implementation research related to osteoarthritis in Asia.

**Methods:**

A systematic and comprehensive search was performed across EMBASE, MEDLINE, Scopus, Web of Science, CINAHL, ScienceDirect, ProQuest, Google Scholar, and Shodhganga, adhering to PRISMA 2020 guidelines. This search was limited to English-language studies. Studies published from 2004 to 2024 focusing on OA implementation research within Asian populations were included. Three reviewers independently performed study selection, data extraction, and quality assessment using the StaRI checklist. A narrative synthesis summarises the findings.

**Results:**

Of 2,365 records identified, seven studies met the inclusion criteria. These studies, conducted across diverse Asian settings, primarily evaluated educational and community-based interventions for osteoarthritis that explicitly reported implementation processes or outcomes. Although reporting quality was generally moderate to high, the studies demonstrated substantial heterogeneity in implementation outcomes, including adherence, fidelity, acceptability, adoption, and effectiveness. Key barriers to implementation included socio-cultural practices, resource constraints, and limitations within healthcare systems.

**Conclusion:**

The scarcity of high-quality implementation studies highlights a significant gap in translating osteoarthritis evidence into practice across Asia. Future research must prioritise context-specific, scalable strategies, standardise outcome measures, and proactively address socio-cultural barriers to optimise osteoarthritis management for the diverse Asian populations.

**Systematic review registration:**

Prospero registration number: CRD42024542237.

## Highlights

- This article systematically reviews implementation research on osteoarthritis in Asia to identify effective strategies and address gaps in translating evidence-based interventions into routine practice
- The review explores various interventions implemented in Asian contexts and then evaluates their effectiveness and outcomes
- The review also investigates the barriers and facilitators that influence the implementation and adoption of osteoarthritis management strategies in Asia

## 1 Introduction

Osteoarthritis (OA) is the third fastest-growing cause of disability worldwide and currently ranks 11th in years lived with disability (1). Affecting nearly 500 million people, OA substantially impairs quality of life, physical functioning, psychological health, and independence (2). The WHO’s Decade of Healthy Ageing (2021–2030) underscores the need to address high-burden chronic conditions such as OA that disproportionately contribute to disability in adults (3, 4). Although often considered a disease of older age, OA frequently begins before 50 years of age, creating opportunities for earlier prevention and intervention (5). If current trends continue, nearly 1 billion people are projected to be living with OA by 2050 (5).

The burden of OA has increased markedly across Asia. According to the Global Burden of Disease 2021 estimates, the age-standardised prevalence of osteoarthritis has increased by 14–18% since 1990, reflecting a sharp escalation in OA among ageing populations (6). Studies from primary care settings across South and Southeast Asia indicate low utilisation of core nonpharmacological strategies, high reliance on analgesics, and limited access to physiotherapy, particularly in rural areas (7–9). These findings highlight substantial implementation gaps and underscore the urgent need for scalable, context-specific OA management approaches across Asia.

Despite robust evidence supporting first-line interventions such as education, physical activity, and weight optimisation, their uptake in routine practice remains suboptimal (10, 11). Only about 35–39% of people with hip or knee OA receive guideline-recommended exercise or education, while many continue to receive low-value, pharmacology-focused care (12). This evidence-practice gap is compounded by inconsistent recommendations across clinical guidelines and their limited applicability to multimorbid patients commonly seen in primary care (13). In low-resource settings, inadequate clinician awareness, fragmented care pathways, poor patient education, and restricted access to allied health services further constrain the delivery of evidence-based OA management (11). At the same time, successful cultural adaptation and implementation of structured self-management programmes such as Good Life with Osteoarthritis from Denmark (GLA: D) in Canada and Australia demonstrate that evidence-based models of OA care can be effectively delivered across diverse health systems, including in Asian contexts (14, 15). These experiences illustrate what is possible when implementation is carefully planned and contextually adapted, underscoring that clinical evidence alone does not change routine practice.

Implementation science provides a systematic approach to understanding and addressing the multilevel determinants of OA care delivery (16–19). It offers tools to identify barriers and facilitators within local health systems, tailor interventions to context, and test strategies such as facilitation, leadership engagement, audit and feedback, and continuous quality monitoring to support sustained integration of evidence-based practices (17, 20, 21). Translating known barriers into practical, scalable solutions remains challenging in settings where a high OA burden coincides with limited health system capacity (22–24).

Strengthening implementation processes and addressing context-specific barriers are critical to expanding access to high-value OA care, optimising limited resources, and ensuring that more individuals benefit from interventions that reduce pain, improve function, and enhance quality of life in rapidly ageing Asian health systems. Yet, the existing evidence on how OA interventions have been implemented, adapted, and scaled within Asian health systems remains fragmented.

Against this backdrop, the present study aims to synthesise existing evidence on implementation research on osteoarthritis in Asia, including implementation strategies, contextual barriers and facilitators, and reported outcomes, to address the question: What is the existing evidence on implementation research on osteoarthritis in Asia?

## 2 Methods

The systematic review protocol was developed adhering to the Preferred Reporting Items for Systematic Reviews and Meta-analyses–Protocol (PRISMA-P) guidelines (25) and prospectively registered in the PROSPERO database (CRD42024542237). The study selection, screening, and reporting processes adhered to the PRISMA 2020 guidelines (26) to ensure methodological rigour, transparency, and reproducibility. All stages of article selection, including duplicate removal, title/abstract screening, and full-text assessment, were conducted using Rayyan software (27) to facilitate blinded and structured decision-making. This review methodology was guided by the PICO (Population, Intervention, Comparator, and Outcome) framework, ensuring a systematic and focused approach to addressing the research question. In line with widely used definitions, “implementation research” is defined as the scientific study of methods and strategies to promote the uptake of evidence-based OA interventions into routine care, and we applied this definition consistently when determining eligibility.

### 2.1 Inclusion and exclusion criteria

The search strategy was systematically adapted for each electronic database to ensure comprehensive and sensitive retrieval of relevant literature. The full eligibility criteria applied in this review are summarised in Table 1. This review included peer-reviewed, English language empirical studies, quantitative, qualitative, or mixed-methods, published between January 2004 and December 2024. Studies were eligible if they were conducted in Asian settings and involved individuals or systems engaged in OA-related implementation research. For the purpose of this review, studies were considered to involve implementation research if they: (i) explicitly aimed to improve, facilitate, or understand the implementation of an evidence-based OA intervention, programme, or strategy; and/or (ii) reported at least one implementation outcome or detailed contextual/process data directly related to how such interventions were integrated into routine care. Only studies that evaluated, applied, or examined the implementation of evidence-based OA interventions, programmes, or strategies were considered for inclusion.

**Table 1:** Inclusion and exclusion criteria.

Category	Inclusion criteria	Exclusion criteria
Population	Individuals of any age with OA or at risk of OA, their caregivers, or healthcare professionals/decision-makers directly involved in OA care, participating in a study with an explicit implementation focus.	Non-human subjects; populations not related to OA care; studies in which OA is not the primary focus of the intervention or implementation effort; or studies where participants are not engaged in OA-related implementation processes (“wrong population”).
Intervention	Any evidence-based intervention with an embedded implementation research component related to OA care in Asian settings. Includes guideline implementation, behavioural interventions, service delivery models, digital health tools, and workforce or organisational strategies, provided that the study examines how these are introduced, scaled up, or sustained in real-world practice.	Interventions that do not involve implementation activities or do not meet the definition of implementation research, including clinical efficacy/effectiveness trials conducted under highly controlled conditions with no explicit implementation aim, and programmes described only in terms of clinical outcomes without implementation strategies or outcomes (“no implementation component”).
Comparator/Control	When applicable, studies with comparator groups such as usual care, alternative implementation strategies, or pre–post conditions were included. The presence of a comparator was not mandatory for eligibility.	Comparator or control conditions outside the scope of implementation-related evaluations or not aligning with OA care pathways.
Outcomes	Studies reporting at least one implementation outcome (e.g., acceptability, adoption, appropriateness, feasibility, fidelity, penetration, sustainability, implementation cost), or providing qualitative or quantitative contextual/process insights that are essential for understanding implementation (e.g., determinants, barriers, and facilitators of implementation).	Outcomes not reporting implementation outcomes and lacking contextual/implementation process information relevant to implementation research (e.g., studies reporting only clinical or epidemiological outcomes without any implementation-related data).
Study design	Empirical quantitative, qualitative, or mixed-methods studies. Quantitative designs included experimental (randomised controlled trials (RCTs) and non-randomised controlled trials (non-RCTs)) and observational designs (quasi-experimental, pre-post, post-only, cross-sectional, cohort, and case–control). Qualitative and mixed-methods studies examining implementation processes or determinants were included.	Non-empirical research, including reviews, systematic reviews, meta-analyses, editorials, commentaries, letters, opinion papers, study protocols, pilot studies without implementation data, case reports/series, surveys without implementation measures, and purely descriptive program documents.
Geographic scope	Asia (based on WHO regional classification), including India.	Studies conducted outside Asia.
Timeframe	2004–2024	Studies published before 2004.

“Wrong population” and “no implementation component” were operationalised *a priori* to enhance consistency and reproducibility. “Wrong population” was applied when the study sample did not primarily consist of: (a) people with OA or at risk of OA, (b) their caregivers, or (c) healthcare professionals, managers, or organisations directly involved in OA care; or when OA was only a minor comorbidity and not the primary focus of the implementation effort. “No implementation component” was applied when a study focused solely on clinical efficacy/effectiveness, epidemiology, or knowledge/attitudes without: (a) an explicit implementation objective, (b) a clearly described implementation strategy, or (c) any implementation outcome or process data as defined above. Studies with a predominant clinical focus were included only when they reported at least one explicit implementation outcome (e.g., adherence, fidelity, adoption) or described structured delivery processes (e.g., session standardisation, reassessment, or feedback mechanisms) relevant to routine clinical integration.

### 2.2 Search strategy (electronic databases)

A preliminary scoping search was first conducted to gain an overview of the existing evidence based on OA-related implementation research. Insights from this initial exploration informed the development of a rigorous and comprehensive search strategy. Subsequently, a comprehensive electronic search was performed in accordance with the Preferred Reporting Items for Systematic Reviews and Meta-Analyses Literature Search Extension (PRISMA-S) guidelines (28), ensuring methodological transparency and reproducibility in identifying relevant studies conducted in Asian settings.

Comprehensive searches were carried out across major electronic databases, including MEDLINE via PubMed, Embase, CINAHL (EBSCO), Scopus, ScienceDirect, ProQuest, and Web of Science. To enhance coverage and reduce publication bias, grey literature sources were also explored through Google Scholar and Shodhganga, with particular attention to dissertations, theses, and institutional reports relevant to OA-related implementation research.

Titles, abstracts, and index terms from key articles were reviewed iteratively to refine and expand the search vocabulary. The final search strategy integrated controlled vocabularies (e.g., MeSH terms, Emtree terms) and free-text keywords, structured around three core domains: (1) Implementation research: implementation science, evidence-based practice, translational research, quality improvement, implementation strategies, process evaluation, barriers, facilitators; (2) Geographic focus (Asia): Asia as a region and individual Asian countries (e.g., India, China, Japan, South Korea, Malaysia, Indonesia etc.); (3) Osteoarthritis: osteoarthritis, knee OA, hip OA, spinal OA, and OA-related interventions.

In addition, the Cochrane Database of Systematic Reviews and PROSPERO were thoroughly searched to identify ongoing or recently completed systematic reviews on related topics, ensuring that the present review addressed existing evidence gaps.

The PRISMA flow diagram summarizing the processes of study identification, screening, eligibility assessment, and final inclusion is provided in Supplementary File S3. Detailed database-specific search syntaxes are available in Supplementary File S1 to facilitate reproducibility.

### 2.3 Study selection and screening process

All records retrieved from the database searches were exported in compatible formats (CSV or RIS) and uploaded into Rayyan QCRI software (27) for efficient reference management. The platform’s automated and manual duplicate-detection functions were used to identify and remove duplicate entries before screening.

A structured two-stage screening procedure was then conducted independently by three reviewers. In the first stage, titles and abstracts were screened against the pre-defined inclusion and exclusion criteria relevant to OA implementation research in Asian settings. Studies considered potentially eligible were retained for further review. Any discrepancies or conflicts among reviewers at this stage were resolved through discussion within Rayyan’s conflict-resolution interface.

In the second stage, full-text articles of all shortlisted studies were retrieved and evaluated in detail using the same eligibility criteria. Operational definitions for key exclusion reasons (e.g., “wrong population,” “no implementation component”, “wrong condition”, “wrong region”) were applied consistently as described in Section 2.1. Reasons for exclusion at the full-text stage were recorded systematically to ensure methodological transparency. Any disagreements during this phase were resolved through consensus, and when necessary, consultation with a designated adjudicator.

### 2.4 Data extraction

Data extraction was conducted using a structured and pre-piloted data extraction form specifically developed for this review. Three reviewers independently extracted information from all eligible studies to ensure accuracy, completeness, and methodological robustness. Extracted data encompassed the following domains:

- Study identification and characteristics: authors, year of publication, country, study setting (e.g., primary care, community, tertiary facility), study design, study period, sample size, target population, and recruitment details.
- Osteoarthritis specific information: type and anatomical site of OA (e.g., knee, hip, spine, generalised), severity classification (where applicable), and population characteristics relevant to OA.
- Intervention characteristics: description of the evidence-based intervention being implemented, underlying rationale, delivery mode, intensity, duration, and personnel involved.
- Implementation strategy: type, description, and theoretical underpinning of implementation strategies used, linked to established taxonomies [e.g., Expert Recommendations for Implementing Change (ERIC), behaviour change approaches] where applicable.
- Contextual determinants: settings, organisational factors, health-system influences, and sociocultural considerations that shaped implementation processes, mapped to an established determinant framework [e.g., Consolidated Framework for Implementation Research (CFIR) domains/constructs]
- Implementation outcomes: extracted verbatim and categorised according to established implementation outcome frameworks.
- Barriers and facilitators: factors influencing implementation processes at individual, organisational, and system levels, mapped to CFIR domains/constructs

CFIR domains and ERIC strategies were applied retrospectively during synthesis to aid interpretation; formal prospective coding was not undertaken due to heterogeneous reporting.

A preliminary pilot extraction using a subset of included studies was conducted to refine the extraction form, harmonise reviewer interpretation of variables, and ensure clarity of the extraction process. Following independent extraction, data were compared across reviewers, and discrepancies were resolved through discussion or adjudication by a senior reviewer to ensure alignment and reliability.

### 2.5 Quality appraisal and risk of bias assessment

Quality appraisal was conducted using the Standards for Reporting Implementation Studies (StaRI) checklist (29), a framework specifically designed to evaluate the methodological and reporting rigour of implementation research. Two reviewers independently assessed each included study against core StaRI domains: (i) articulation of implementation aims, (ii) adequacy of intervention and implementation strategy descriptions, (iii) reporting of contextual and system-level determinants, (iv) documentation of fidelity, adaptations, and implementation processes, (v) specification and measurement of implementation outcomes, and (vi) overall reporting transparency and coherence.

The StaRI checklist consists of 27 items. Each item was coded using a binary scoring system (“1” = reported; “0” = unreported). This structured approach facilitated the systematic identification of limitations related to incomplete reporting, including incomplete intervention descriptions, insufficient contextual detail, unclear implementation processes, and poor reporting of outcomes. After independent scoring, reviewer assessments were compared to determine inter-rater consistency. Discrepancies were resolved through deliberation, and where disagreement persisted, a third senior reviewer provided adjudication.

In addition to StaRI-based appraisal, the risk of bias was assessed using study design–appropriate tools. Randomised controlled trials were evaluated using the Cochrane Risk of Bias 2 (RoB 2) tool (30), including the cluster-randomised trial extension where applicable. Non-randomised studies were assessed using the ROBINS-I tool (30), and mixed-methods studies were appraised using the Mixed Methods Appraisal Tool (MMAT) (31). Risk-of-bias assessments were conducted independently by two reviewers. Disagreements were resolved through discussion; where consensus could not be reached, a third senior reviewer adjudicated.

### 2.6 Data synthesis

Data synthesis was guided by the Synthesis Without Meta-analysis (SWiM) reporting guideline (32). Given the considerable heterogeneity across included studies, including variation in intervention types, implementation strategies, contextual determinants, methodological approaches, and outcome measures, a quantitative meta-analysis was not feasible.

A structured narrative synthesis was therefore conducted. The synthesis process involved:

- Grouping studies by intervention type (e.g., exercise therapy, education programs, self-care programs, clinical practice guidelines)
- Comparing implementation strategies and mapping them to established frameworks (e.g., ERIC strategies, behaviour change techniques)
- Identifying contextual determinants influencing implementation success or failure across diverse Asian health-care settings
- Synthesising implementation outcomes, noting variability in definitions, measurement tools, and reporting quality
- Thematic analysis of barriers and facilitators at individual, provider, organisational, and system levels
- Identifying gaps and inconsistencies, including under-reported outcomes, limited fidelity assessment, or challenges in sustaining interventions

Reported determinants and strategies were narratively compared with CFIR domains and ERIC strategy categories to support interpretive synthesis, rather than formal framework-based coding. The narrative synthesis was conducted iteratively, with findings cross-checked against extracted data to ensure accuracy.

## 3 Results

The search yielded 2,365 records, of which 2,151 records were screened after removing duplicates. Twenty full-text articles were assessed, and 13 were excluded: eight for lacking an implementation component and five for evaluating non-OA-specific populations (e.g., rheumatoid arthritis, chronic low back pain, general geriatric mobility programs, or perioperative orthopaedic pathways unrelated to non-surgical OA care). Seven studies met all inclusion criteria (Supplementary File S3), with key characteristics summarised in Table 2.

**Table 2:** Characteristics of included studies.

Author	Country	Types of osteoarthritis	Study designs	Purpose	Intervention	Implementation strategies	Implementation outcomes	Result
Goh et al. (33)	Singapore	Knee	–	To increase the competency of specialist outpatient clinic nurses in the provision of pre-operative TKR education	Education package	(1) Develop a teaching plan (2) Conduct TKR pre-op education for clinic staff nurses (3) Allocate a timetable to train clinic nurses (4) Assess the competency of staff nurses post-training (5) Develop a standardized TKR Patient Pre-op Education Package, inclusive of video (6) Provide training facilities such as a patient education room, an LCD screen and DVD player for TKR video	Adoption Acceptability Fidelity	(1) The project improved nurses’ competency in the provision of pre-operative TKR education (increased from 18% to 91%) (2) The percentage of patients who received pre–operative TKR education increased from 27% to 80% (3) The proportion of patients who received the structured pre-operative education package increased from 27% to 50%. (4) The mean length of hospital stay decreases from 9.64 to 7.66 days.
Opava et al (34)	India	Hip and Knee	Mixed-method	To describe Indian PTs’ knowledge, attitudes, and confidence on evidence‐ based management of OA.	Swedish national initiative “Better Management of Patients with OA” (BOA) model	(1) Training and education of BOA (2) Trained individuals with OA share their experiences and facilitate education for PTs (3) Identifying barriers through FGDs (4) Identifying barriers (5) Creating an Indian BOA	Acceptability Adherence	(1) 74% of PTs agreed that radiography determines the type of treatment required, (2) 69% agreed that a prescription for exercise is enough to ensure adherence. (3) Confidence in guiding the physical activity was generally high (≥5 on 6‐point scales).
Thapa et al (36)	Nepal	Knee and Hip	Cluster randomized trial	To evaluate the effectiveness of pharmacist-led educational intervention and medication review among osteoarthritis patients	(1) Educational intervention Medication review	(1) Regular visits, phone calls, and educational materials to deliver education reviews, (2) Regular follow-up calls to clarify doubts, and the use of multimedia educational materials	Acceptability Adherence Fidelity Adoption	(1) Significant improvements in pain scores and knowledge physical function, depression, or quality of life (3) An improvement in knowledge scores was observed in the intervention group at 3 months
Aree-Ue et al (35)	Thailand	Knee	Quasi-experimental	To examine the patient outcomes of a comprehensive health education plus village health volunteer monitoringsupport	(1) Comprehensive health education (2) Village health volunteer monitoring support program	(1) Comprehensive health education plus monitoring support from village health volunteers	Adherence Fidelity Acceptability	Significant improvements in pain, physical ability, depressive symptoms, fatigue, quality of life, and sleep quality
Batra et al (37)	India	Knee	–	To evaluate the influence of 2 testing positions (sitting versus prone lying) on proprioceptive knee assessment score in patients with early knee osteoarthritis	(1) Context-specific proprioceptive retraining (2) Multi-joint coupling strategies (3) Conventional treatment	(1) Standardised Exercise Sessions (2) Periodic reassessment of proprioceptive acuity (3) Real-time feedback to patients during exercises to support correct execution and adherence	Adherence (session completion) Fidelity (protocol-based delivery) Adoption (integration into physiotherapy practice)	Group 1 The changes in proprioceptive acuity scores of the left knee at 30° (*p* = 0.004), the left knee at 60° (*p* = 0.002), and the right knee at 60° (*p* < 0.001) were statistically significant Group 2 The changes in proprioceptive acuity scores of the left knee at 30° (*p* = 0.004), the left knee at 60° (*p* = 0.028), and the right knee at 60° (*p* = 0.009) were statistically significant.
Lee et al (38)	Hong Kong	Knee	Mixed-method	This pilot study estimated the effects of a tailor-made exercise program on exercise adherence and health outcomes, and explored the participants’ Perception and experience of the program	Community-based group exercise program	(1) Participants practiced the exercises in class with corrections provided, which helped them master the movements. (2) Participants were given materials to assist them in practicing the exercises at home. (3) Scheduling follow-up sessions within the first few months to ensure safety and reinforce correct exercise movements.	Adherence, Acceptability	(1) 91.04%–overall high adherence level to the recommended exercise practice frequency. (2) 76.71%–overall good performance in mastering the exercise movements (3) (43.2% for pain, 43.5% for stiffness, and 50% for physical functioning) and total scores (45.9%) of WOMAC, the mental health summary score of SF-12 (16.3%), the degree of ROM (13.5%), and the reduction in time required for TST (15.8%). (4) The participants' satisfaction score – an overall high level of satisfaction with the exercise program.
Lee et al (39) (2012)	South Korea	Not specified	Randomized trial	To evaluate a self-care program for elders with osteoarthritis managed by primary health care workers, and community health practitioners (CHPs) in rural Korea.	Self-care program (SCP)	Rural Korea	Adherence Fidelity Adoption	(1) 6-week SCP for arthritis managed by primary health care workers (CHP at community health post) were effective at reducing pain and increasing the self-care ability

Although pharmacological, physical therapy, lifestyle, and traditional medicine interventions were eligible, no studies evaluating these approaches reported implementation processes or outcomes. Their exclusion underscores a broader gap in osteoarthritis implementation research within Asian contexts, rather than limitations of the search strategy.

The seven included studies, conducted between 2011 and 2024 across various Asian settings, employed mixed-methods, randomised controlled, quasi-experimental, or cluster-randomised designs. All studies delivered a clinical intervention supported by at least one implementation strategy, although most did not explicitly describe these strategies using implementation science terminology.

### 3.1 Clinical interventions and implementation strategies

Interventions were grouped into: (a) clinical components—what was delivered, and (b) implementation strategies—activities intended to support delivery, uptake, and sustainability.

Clinical interventions included: Educational interventions (*n* = 4): lectures, printed materials, videos, or multimedia modules to improve OA knowledge, self-management skills, or provider competencies (33–36); Exercise programs (*n* = 2): proprioceptive or community-based activity interventions designed for older adults (37, 38); Self-care management program (*n* = 1): a community health practitioner–led model focused on strengthening self-efficacy and symptom management (39).

Implementation strategies, broadly aligned with ERIC categories, included provider training and capacity building, structured follow-up or reminder systems, community mobilisation and peer support, audit and feedback, and multimedia-based dissemination. Only two studies explicitly named these components as “implementation strategies”; in others, they were embedded within intervention delivery without a stated theoretical rationale or guiding framework.

### 3.2 Implementation outcomes

All seven included studies assessed at least one implementation outcome consistent with Proctor et al.’s framework (40), though definitions and measurement methods varied across studies. Table 3 maps each study to eight key implementation outcomes: acceptability, adoption, appropriateness, feasibility, adherence, fidelity, penetration, and sustainability. The major implementation outcomes identified are presented below.

- Adherence [*n* = 5, i.e., Opava et al. (34), Aree-Ue et al. (35), Thapa et al. (36), Batra et al. (37), Lee et al. (39)]: assessed through attendance logs, participant diaries, or provider checklists
- Fidelity [*n* = 4, i.e., Opava et al. (34), Aree-Ue et al. (35), Thapa et al. (36), Batra et al. (37), Lee et al. (39)]: measured through facilitator checklists or structured observations
- Acceptability [*n* = 3, i.e., Opava et al. (34), Aree-Ue et al. (35), Thapa et al. (36), Batra et al. (37), Lee et al. (39)]: captured via satisfaction surveys or qualitative interviews, with participants and providers
- Adoption [*n* = 2, i.e., Thapa et al. (36), Lee et al. (39)]: reflected through indicators of initial uptake or sustained use in routine practice, such as provider willingness to continue program delivery or maintain relevant tools.

**Table 3:** Mapping of included studies to implementation outcomes.

Study (Country)	Intervention type	Acceptability	Adoption	Appropriateness	Feasibility	Adherence	Fidelity	Penetration	Sustainability
Opava et al. (34) (India, adapted from Sweden)	BOA educational model	Patient and provider acceptability after cultural adaptation	In select sites	Aligned with patient needs and local culture	Limited by resource constraints	Present	Variable adherence to BOA protocols	Implemented in few centers	Unclear — follow-up data limited
Thapa et al. (36) (Nepal)	Community-based education and follow-up	Community welcomed sessions	In target communities	Addressed mobility and cultural needs	Good feasibility in rural setting	Most participants attended sessions	Consistent delivery	Engaged several villages	Unclear — no long-term data
Aree-Ue et al. (35) (Thailand)	Education with community involvement	Present	By local leaders and patients	Cultural appropriateness	Feasible with local health worker support	Majority completed sessions	Structured materials ensured fidelity	Penetration in community	Short-term continuation reported; long-term sustainability not assessed
Goh et al. (33) (Singapore)	Pre-op TKR education	Present (for patients and nurses)	In surgical units	Present	Feasibility limited by time and staffing constraints	Variable patient engagement	Inconsistent nurse delivery	Limited to single hospital	Sustainability threatened by staff turnover
Lee et al. (38) (Hong Kong)	Group exercise program	Participants valued sessions	Only some eligible adults joined	Present	Feasibility limited by need for space, staff	Attendance variable	Protocol deviations	Limited penetration	Ceased post-study due to no funding
Lee et al. (39) (South Korea)	Self-care via CHPs	Community-trusted CHPs	CHPs integrated program into practice	Appropriateness for low-access areas	Good feasibility with training	Most participants completed program	Supervision ensured adherence	Penetration limited to participating rural communities during study period	Continuation dependent on external support
Batra et al. (37) (India)	Proprioceptive retraining + manual therapy	Participants engaged well	In physiotherapy units	Present	Feasible in clinical settings	Most participants completed sessions	Structured protocol ensured fidelity	Limited to urban clinics	Unclear — no long-term tracking

*“Present” indicates that the implementation outcome could be clearly inferred from study descriptions. Penetration and sustainability were infrequently reported, and where noted, were based on short-term implementation scopes or continuation during the study period; no study reported long-term follow-up (≥12 months).

One study also reported clinical improvements (e.g., pain reduction and enhanced self-care ability) as indicators of implementation-linked effectiveness. Interpretation of penetration and sustainability outcomes should be treated cautiously, as these were largely inferred from short-term implementation scope and author statements rather than measured through longitudinal follow-up. Although none of the studies explicitly applied the CFIR framework, several reported contextual and process-related factors that aligned conceptually with CFIR domains, including intervention simplicity and adaptability, provider knowledge and capacity, participant health literacy, and process factors such as training quality and engagement. These contextual factors likely influenced variability in implementation outcomes.

Overall, studies demonstrated strong participant engagement and consistent delivery of the intervention. However, substantial heterogeneity in outcome definitions, measurement tools, and reporting formats limited the comparability of implementation outcomes across studies.

### 3.3 Quality appraisal and risk of bias assessment

#### 3.3.1 Reporting quality (StaRI)

The StaRI checklist was used to appraise the completeness and transparency of reporting across the seven included studies, with separate scores assigned to the implementation and intervention domains (Supplementary File S2: StaRI checklist). Total StaRI scores (out of 27) were summarized using the following descriptive bands to indicate reporting completeness: high completeness (≥20 items), moderate completeness (15–19 items), and low completeness (<15 items).

- High reporting completeness: Four studies [Goh et al. (33), Opava et al. (34), Aree-Ue et al. (35), Thapa et al. (36)] scored ≥20 in at least one domain, reflecting comprehensive reporting of interventions, implementation strategies, and contextual factors.
- Moderate reporting completeness: Batra et al. (2010) (37), Lee et al. (38), and Lee et al. (39) scored 15–19, mainly due to incomplete description of implementation strategies, contextual determinants, and theoretical foundations.
- Low reporting completeness: None of the studies scored <15, indicating that no study had critically poor reporting.

#### 3.3.2 Domain-specific observations (StaRI)

- Implementation domain: Scores ranged from 13 to 21. Lee et al. (38) had the lowest score (13), reflecting minimal explicit description of implementation processes despite well-reported intervention outcomes, whereas Goh et al. (33) and Opava et al. scored highest (21), demonstrating detailed reporting of implementation strategies, follow-up procedures, and contextual considerations.
- Intervention domain: Scores ranged from 15 to 21, indicating generally moderate-to-high reporting of clinical intervention components. All studies adequately described intervention content, delivery methods, and target populations, although fewer integrated intervention reporting with implementation rationale or theoretical frameworks.

The reporting appraisal underscores the importance for future OA implementation studies in Asia to adhere to reporting standards such as StaRI, particularly for implementation processes, theoretical foundations, and context-specific adaptations to enhance transparency and reproducibility.

#### 3.3.3 Risk of bias assessment (design-aligned tools)

##### 3.3.3.1 Randomised controlled trials (RoB 2)

Risk of bias in randomised controlled trials was assessed using the Cochrane RoB 2 tool at the outcome level. Eight outcomes from three RCTs (cluster and parallel designs) were evaluated (Tables 4, 5). No outcome was judged to be at low risk of bias; six outcomes (75.0%) were rated as having some concerns, and two (25.0%) as high risk of bias (Table 4). Both outcomes from Yul et al. were judged at high risk of bias, driven by concerns regarding deviations from intended interventions, lack of blinding, and outcome measurement, with bias favouring the experimental intervention. All six outcomes from Thappa et al. were judged to have some concerns, reflecting uncertainties in randomisation, outcome measurement, and missing data, and were deemed to favour the experimental intervention. The outcome from Batra et al. was judged as having some concerns, with an unpredictable direction of bias. Overall, bias was judged to favour the experimental intervention for 87.5% of outcomes, with no outcome judged to favour the comparator.

**Table 4:** Outcome-level RoB 2 overall risk-of-bias judgements and predicted direction of bias for randomized controlled trials (*n* = 8 outcomes).

Study	Trial type	Outcome	Overall RoB 2 judgement	Direction of bias
Lee et al. (39)	Cluster RCT	Number of painful joints	High	Favours experimental
		Arthritis management skill (Likert)		
Thapa et al. (36)	Cluster RCT	Pain intensity (NRS)	Some concerns	Favours experimental
		Physical function (WOMAC)		
		OA knowledge score		
		PROMIS Depression 8b		
		Quality of life (EQ-5D / EQ-VAS)		
Batra et al. (37)	Parallel RCT	Proprioceptive acuity	Some concerns	Unpredictable

**Table 5:** Cross-tabulation of RoB 2 overall risk-of-bias judgements and predicted direction of bias across outcomes (*n* = 8).

Overall RoB 2 judgement	Favours experimental *n* (%)	Unpredictable *n* (%)	Favours comparator *n* (%)	Total *n* (%)
Low risk	0 (0.0)	0 (0.0)	0 (0.0)	0 (0.0)
Some concerns	6 (75.0)	0 (0.0)	0 (0.0)	6 (75.0)
High risk	1 (12.5)	1 (12.5)	0 (0.0)	2 (25.0)
Total	7 (87.5)	1 (12.5)	0 (0.0)	8 (100.0)

##### 3.3.3.2 Non-randomised studies (ROBINS-I)

The risk of bias in non-randomised studies was assessed at the study level using the ROBINS-I tool (Tables 6, 7). All three studies [Goh et al. (33), Aree-Ue et al. (35), Lee et al. (38)] were judged to be at serious risk of bias, primarily due to confounding, participant selection, the absence of control groups, and limited adjustment for prognostic factors. Additional concerns were noted in the domains of outcome measurement and deviations from intended interventions, particularly where outcomes were self-reported, and blinding was not feasible. For all studies, bias was judged to favour the intervention.

**Table 6:** Study-level ROBINS-I overall risk-of-bias judgements and direction of bias for non-randomized studies (*n* = 3 studies).

Study	Design	ROBINS-I overall judgement	Direction of bias
Goh et al. (33)	Before–after (non-randomized)	Serious risk	Favours intervention
Lee et al. (38)	Single-group pretest–posttest	Serious risk	Favours intervention
Aree et al. (35)	Non-randomized	Serious risk	Favours intervention

**Table 7:** Cross-tabulation of ROBINS-I overall risk-of-bias judgements and predicted direction of bias across studies (*n* = 3).

Overall ROBINS-I judgement	Favours intervention *n* (%)	Unpredictable *n* (%)	Favours comparator *n* (%)	Total *n* (%)
Low risk	0 (0.0)	0 (0.0)	0 (0.0)	0 (0.0)
Moderate risk	0 (0.0)	0 (0.0)	0 (0.0)	0 (0.0)
Serious risk	3 (100.0)	0 (0.0)	0 (0.0)	3 (100.0)
Critical risk	0 (0.0)	0 (0.0)	0 (0.0)	0 (0.0)
Total	3 (100.0)	0 (0.0)	0 (0.0)	3 (100.0)

##### 3.3.3.3 Mixed-methods study (MMAT)

The mixed-methods study by Opava et al. was appraised using MMAT (2018), which discourages numerical scoring (Table 8). The study met screening criteria, with clearly stated research questions and appropriate qualitative and quantitative data. The qualitative component was methodologically sound, while the quantitative, non-randomised component partially met the criteria due to the absence of a control group and unaddressed confounding factors. Mixed-methods integration was mostly met, with limited discussion of divergences. Overall, the study was judged to have moderate methodological quality, with reduced confidence in quantitative findings.

**Table 8:** Mixed methods appraisal tool (MMAT) assessment of methodological quality in Opava et al. (34).

MMAT component	Appraisal outcome	Key methodological considerations
Screening questions	Met	Research questions were clearly stated, and the collected qualitative and quantitative data were appropriate to address the study objectives
Qualitative component	Met	Qualitative approach, data collection, analysis, and interpretation were appropriate and coherent, with findings adequately supported by participant quotations
Quantitative non-randomised component	Partially met	Outcome measures were appropriate, and data completeness was adequate; however, the absence of a control group and the lack of adjustment for confounding limit internal validity
Mixed-methods integration	Mostly met	Clear rationale for a mixed-methods design and integration of qualitative and quantitative findings at the interpretation stage; limited explicit discussion of divergences
Overall MMAT judgement	Moderate methodological quality	Confidence in findings is reduced primarily by limitations in the quantitative non-randomised component, particularly unaddressed confounding

*The MMAT does not recommend numerical scoring; therefore, an overall qualitative judgement of methodological quality is presented.

## 4 Discussion

This systematic review synthesised evidence from seven studies evaluating OA interventions implemented across diverse Asian contexts over a 13-year period. The limited number of studies highlights a substantial gap in the application of implementation science principles to OA management in the region. Most studies focused on knee OA and were conducted in varied settings, including clinics, community health centres, and rural areas. Interventions included educational programs, group exercise, self-care programs, and context-specific proprioceptive retraining. Key implementation outcomes reported included adherence, fidelity, acceptability, adoption, and sustainability; however, heterogeneity in outcome definitions and measurement limited cross-study comparability. Common barriers included cultural norms, limited resources, and inconsistent reporting of implementation strategies and contextual determinants.

### 4.1 Educational interventions

Several studies have applied training and education strategies to improve the capacity of providers and patients to manage OA. For example, Opava et al. described the Swedish “Better management of patients with OA” (BOA) model, which was adapted to the Indian context using cultural tailoring and stakeholder engagement (34). This adaptation demonstrated good acceptability and adherence, despite barriers such as entrenched cultural practices and insufficient systemic support. This aligns with the findings of Aree-Ue et al. (35) and Thapa et al. (36) on community involvement and continuous follow-up as crucial facilitators for promoting intervention uptake. These findings underscore the observation in the result section that, while adherence and acceptability were generally high, explicit reporting of implementation strategies and contextual determinants was often lacking, which limits replicability. The pre-operative education package for Total Knee Replacement (TKR) developed by Goh et al. in Singapore substantially improved nurse competency and patient education rates, despite barriers such as inconsistent knowledge and time constraints (33). These studies emphasise that region-specific implementation strategies must be embedded with ongoing training, multi-level stakeholder engagement, and continuous quality monitoring to sustain impact.

### 4.2 Community-based group exercise program

Lee et al. implemented a community-based group exercise program for older adults with knee OA in Hong Kong, while culturally tailored interventions improved adherence and health outcomes, the lack of structured implementation protocols, fidelity checklists, and process monitoring limited sustainability (38). This aligns with our Results, which showed variability in fidelity measurement and outcome definitions across studies, highlighting the need for standardised reporting and context-sensitive implementation planning. The involvement of community health workers and integration of refresher sessions may strengthen adoption and long-term maintenance. The lack of consistent measurement of implementation outcomes, such as acceptability, feasibility, and sustainability, limits cross-study comparison and the accumulation of transferable lessons.

### 4.3 Self-care program

The study by Yul et al. evaluated a 6-week self-care program delivered by trained Community Health Practitioners (CHPs), which demonstrated significant improvements in pain management and arthritis self-care (39). This aligns with our review’s findings on the importance of community-based, patient-centred OA interventions. The successful integration of CHPs and a self-care model offers a valuable and adaptable framework for other Asian contexts, particularly in areas with limited access to healthcare. Consistent with our review’s results, successful implementation relied on provider capacity, quality training, and community engagement, consistent with CFIR domains. Future efforts should prioritise comprehensive training, clear role definitions, and ongoing supervision to maintain fidelity, sustainability, and community ownership.

### 4.4 Context-specific proprioceptive retraining along with conventional treatment

Batra et al. demonstrated that the prone lying position was significantly more effective than sitting for assessing proprioceptive acuity in patients with early knee osteoarthritis, showing statistically significant improvements in scores on post-treatment (37). Their implementation strategies included a structured educational package delivered three times a week over 8 weeks. This package encompassed context-specific adaptation and multi-component implementation strategies (combining patient education, proprioceptive retraining, and multi-joint coupling strategies, utilising targeted exercises and manual therapy) to maximise clinical outcomes and implementation feasibility (37). High participant acceptability and adherence mirrored the positive implementation outcomes reported in our Results; however, structured implementation planning was essential to achieve consistent fidelity, highlighting the need to integrate clinical effectiveness with practical implementation considerations.

## 5 Limitations

This review has several limitations. It may under-represent digital and telehealth-based implementation strategies for osteoarthritis management in Asia. Although telehealth interventions are increasingly utilised in several Asian countries, particularly following the COVID-19 pandemic, many such initiatives may not yet have been formally evaluated or published in peer-reviewed journals. First, the review was restricted to peer-reviewed English-language literature, which may disproportionately undercount Asia-based implementation research, as service delivery innovations are often reported in local-language journals and in grey literature (e.g., government/NGO reports, program evaluations, theses, and conference proceedings) rather than in indexed English journals. As grey literature was not searched, locally implemented or scaled models of care, including digital and telehealth initiatives, may be under-represented, which could bias the evidence toward settings more likely to publish in English and limit the completeness and generalizability of our findings across Asian health systems. Second, substantial heterogeneity in intervention types, study contexts, and outcome definitions hindered synthesis and precluded meta-analysis. Third, inconsistent reporting of implementation strategies and outcomes, as highlighted in the Results, limits cross-study comparability and the generalisability of findings beyond similar Asian settings. Because CFIR and ERIC frameworks were applied retrospectively and descriptively, rather than through formal coding, interpretations of determinants and strategies should be considered exploratory. Most studies lacked longitudinal follow-up, which limited the robust assessment of sustainability and penetration; consequently, these outcomes were inferred rather than empirically confirmed. Additionally, the limited number of included studies restricted our ability to examine how contextual differences across regions influence implementation success. Finally, relevant implementation studies published before 2004 or after 2024 may not have been captured due to the predefined search window specified in the protocol. As implementation science in OA is a rapidly evolving field, newer studies may provide additional insights beyond those synthesised here.

## 6 Implications for implementation research and practice

The findings of this review have several implications for implementation research in osteoarthritis care within Asian contexts. First, while most included studies demonstrated favourable adherence, fidelity, and acceptability, these outcomes were more consistently reported than higher-order implementation outcomes such as penetration and sustainability. The latter were frequently inferred from short-term continuation or author statements, rather than measured using longitudinal or system-level indicators, which limited confidence in conclusions regarding long-term integration into routine care.

Second, variation in implementation-domain reporting, reflected in differing StaRI implementation scores, suggests that some studies prioritized clinical effectiveness over explicit documentation of implementation processes. This uneven reporting necessitated the use of qualitative, study-specific categorizations of implementation outcomes, which constrain cross-study comparability. Future studies should incorporate standardized implementation frameworks (e.g., CFIR, ERIC, Proctor outcomes) prospectively and define explicit metrics for adoption, penetration, and sustainability.

Finally, the predominance of facility-based and community-delivered interventions, with limited representation of digital or telehealth strategies, highlights a potential gap in the current literature. Given the expanding role of digital health in Asia, future implementation studies should evaluate technology-enabled models of osteoarthritis care and report implementation outcomes using consistent, theory-informed approaches to strengthen the translation of evidence.

## 7 Conclusion and recommendation

Osteoarthritis is a significant cause of disability in Asia, yet despite established evidence-based treatments, its uptake in routine care remains poor. Our review identified only seven implementation studies in the region, primarily educational in focus, with methodological strengths but persistent challenges, including cultural norms, resource constraints, and inconsistent measurement of outcomes. This scarcity of region-specific, high-quality research underscores the urgent need to identify effective, culturally tailored, and resource-sensitive implementation strategies. Our study addresses this gap by systematically examining existing interventions to inform policymakers and guide the integration of implementation science principles into OA management across diverse Asian settings.

## Data Availability

The original contributions presented in the study are included in the article/Supplementary material, further inquiries can be directed to the corresponding author.
