# Supplementary material for: Implementation research on osteoarthritis in Asia: a systematic review
Source: Front Public Health. 2026 Feb 10;14:1693849. doi: 10.3389/fpubh.2026.1693849 (PMC12931281; doi:10.3389/fpubh.2026.1693849)
Supplement: Supplementary file 1 [file Table_1.DOCX]

| **Search Strategy**  **Concepts**  **(Key words)** | **Osteoarthritis**  **OA** | **Implementation research** | **ASIA** |
| --- | --- | --- | --- |
| **MeSH Terms** | "Osteoarthritis"[Mesh] OR  "Osteoarthritis, Spine"[Mesh] OR  "Osteoarthritis, Knee"[Mesh] OR  "Osteoarthritis, Hip"[Mesh] OR | "Implementation Science"[Mesh] OR  "Health Plan Implementation"[Mesh] | "Asia"[Mesh] |
| **Text words** | **“Osteoarthritis" OR "Osteoarthrosis" OR "osteoarthritides" OR "arthritis degenerative" OR "degenerative arthritis" OR "arthros*" OR "osteoarthrosis deformans" OR "osteoarthritis of spine" OR "spine osteoarthritis" OR "spinal osteoarthritis" OR "osteoarthritis spinal" OR "osteoarthritis of the spine" OR "lumbar osteoarthritis" OR "osteoarthritis lumbar" OR "knee osteoarthritis" OR "osteoarthritis of knee" OR "hip osteoarthritis" OR "Coxarthrosis" OR "Coxarthroses" OR "osteoarthritis of the hip*" OR "degenerative arthritis" OR "degenerative joint disease" OR "ankle osteoarthritis" OR “Wear-and-tear arthritis” OR “Joint degeneration” OR “Cartilage degradation” OR “Bone spurs” OR “Osteophytes” OR “Joint stiffness” OR “Crepitus” OR “Joint inflammation” OR “Joint effusion” OR “Hand osteoarthritis” OR “**Shoulder Osteoarthritis” OR “Physical Therapy for Osteoarthritis” OR “Inflammatory Mediators in Osteoarthritis” OR “Osteoarthritis Clinical Trials” | “Implementation Science*” OR “Health Plan Implementation*” OR Implementation OR implementing OR implemented OR operations OR delivery OR deliver OR “translational science” OR “translational research” OR “Translational Medical Research” OR “quality improvement” OR “task shifting” OR policy OR monitoring OR evaluation OR “implementation process evaluation” OR “impact evaluation” OR “effectiveness, implementation” OR “hybrid research” OR “applied implementation research” OR “implementation studies” OR “applied research” OR “practice based research” OR “operational research” OR “applied implementation science” OR “outcome evaluation” OR “evidence based practice” OR “policy implementation” OR “stakeholder engagement” OR “knowledge translation” OR “contextual factors” OR “barriers and facilitators” OR “implementation strategies” OR “implementation outcomes” OR “real world implementation” OR “implementation framework” OR “Implementation fidelity” OR “Implementation interventions” OR “Implementation research methods” OR “Translation of research into practice” | **Asia OR "British Indian Ocean Territory" OR Bangladesh OR India OR Bharat OR "Republic of India" OR "Khmer Republic" OR Cambodia OR Kampuchea OR "Republic of China" OR "Mainland China" OR "Netherlands East Indies" OR "East Indies" OR "West Irian" OR "New Guinea, Indonesian" OR "New Guinea, West" OR "Indonesian New Guinea" OR "Irian Jaya" OR "Federal Democratic Republic of Nepal" OR "Islamic Republic of Pakistan" OR Phillipines OR Phillippines OR Philipines OR "Kingdom of Thailand" OR Siam OR "Viet Nam" OR "Vietnam, Republic of" OR "North Vietnam" OR Srilanka** |

**Supplementary file S1: Search strategy**

**Title:** Implementation research on osteoarthritis in Asia: A systematic review

**PUBMED – Total no of hits - 351**

| **Serial no** | **Search terms** | **No of hits** |
| --- | --- | --- |
| #1 | **("Implementation Science"[Mesh] OR "Health Plan Implementation"[Mesh]) OR ("Implementation Science*"[Title/Abstract] OR "Health Plan Implementation*"[Title/Abstract] OR Implementation[Title/Abstract] OR implementing[Title/Abstract] OR implemented[Title/Abstract] OR operations[Title/Abstract] OR delivery[Title/Abstract] OR deliver[Title/Abstract] OR "translational science"[Title/Abstract] OR "translational research"[Title/Abstract] OR "Translational Medical Research"[Title/Abstract] OR "quality improvement"[Title/Abstract] OR "task shifting"[Title/Abstract] OR policy[Title/Abstract] OR monitoring[Title/Abstract] OR evaluation[Title/Abstract] OR "implementation process evaluation"[Title/Abstract] OR "impact evaluation"[Title/Abstract] OR "effectiveness, implementation"[Title/Abstract] OR "hybrid research"[Title/Abstract] OR "implementation studies"[Title/Abstract] OR "applied research"[Title/Abstract] OR "practice based research"[Title/Abstract] OR "operational research"[Title/Abstract] OR "applied implementation science"[Title/Abstract] OR "outcome evaluation"[Title/Abstract] OR "evidence based practice"[Title/Abstract] OR "policy implementation"[Title/Abstract] OR "stakeholder engagement"[Title/Abstract] OR "knowledge translation"[Title/Abstract] OR "contextual factors"[Title/Abstract] OR "barriers and facilitators"[Title/Abstract] OR "implementation strategies"[Title/Abstract] OR "implementation outcomes"[Title/Abstract] OR "real world implementation"[Title/Abstract] OR "implementation framework"[Title/Abstract] OR "Implementation fidelity"[Title/Abstract] OR "Implementation interventions"[Title/Abstract] OR "Implementation research methods"[Title/Abstract])** | 36,98,214 |
| #2 | **("Osteoarthritis"[Mesh] OR "Osteoarthritis, Spine"[Mesh] OR "Osteoarthritis, Knee"[Mesh] OR "Osteoarthritis, Hip"[Mesh]) OR ("Osteoarthritis"[Title/Abstract] OR "Osteoarthrosis"[Title/Abstract] OR "osteoarthritides"[Title/Abstract] OR "arthritis degenerative"[Title/Abstract] OR "degenerative arthritis"[Title/Abstract] OR "arthros*"[Title/Abstract] OR "osteoarthrosis deformans"[Title/Abstract] OR "osteoarthritis of spine"[Title/Abstract] OR "spine osteoarthritis"[Title/Abstract] OR "spinal osteoarthritis"[Title/Abstract] OR "osteoarthritis spinal"[Title/Abstract] OR "osteoarthritis of the spine"[Title/Abstract] OR "lumbar osteoarthritis"[Title/Abstract] OR "osteoarthritis lumbar"[Title/Abstract] OR "knee osteoarthritis"[Title/Abstract] OR "osteoarthritis of knee"[Title/Abstract] OR "hip osteoarthritis"[Title/Abstract] OR "Coxarthrosis"[Title/Abstract] OR "Coxarthroses"[Title/Abstract] OR "osteoarthritis of the hip*"[Title/Abstract] OR "degenerative arthritis"[Title/Abstract] OR "degenerative joint disease"[Title/Abstract] OR "ankle osteoarthritis"[Title/Abstract] OR "Wear-and-tear arthritis"[Title/Abstract] OR "Joint degeneration"[Title/Abstract] OR "Cartilage degradation"[Title/Abstract] OR "Bone spurs"[Title/Abstract] OR "Osteophytes"[Title/Abstract] OR "Joint stiffness"[Title/Abstract] OR "Crepitus"[Title/Abstract] OR "Joint inflammation"[Title/Abstract] OR "Joint effusion"[Title/Abstract] OR "Hand osteoarthritis"[Title/Abstract] OR "Shoulder Osteoarthritis"[Title/Abstract] OR "Osteoarthritis Clinical Trials"[Title/Abstract])** | 1,74,580 |
| #3 | **("Asia"[Mesh] OR Asia[Title/Abstract] OR "British Indian Ocean Territory"[Title/Abstract] OR Bangladesh[Title/Abstract] OR India[Title/Abstract] OR Bharat[Title/Abstract] OR "Republic of India"[Title/Abstract] OR "Khmer Republic"[Title/Abstract] OR Cambodia[Title/Abstract] OR Kampuchea[Title/Abstract] OR "Republic of China"[Title/Abstract] OR "Mainland China"[Title/Abstract] OR "Netherlands East Indies"[Title/Abstract] OR "East Indies"[Title/Abstract] OR "West Irian"[Title/Abstract] OR "New Guinea, Indonesian"[Title/Abstract] OR "New Guinea, West"[Title/Abstract] OR "Indonesian New Guinea"[Title/Abstract] OR "Irian Jaya"[Title/Abstract] OR "Federal Democratic Republic of Nepal"[Title/Abstract] OR "Islamic Republic of Pakistan"[Title/Abstract] OR Phillipines[Title/Abstract] OR Phillippines[Title/Abstract] OR Philipines[Title/Abstract] OR "Kingdom of Thailand"[Title/Abstract] OR Siam[Title/Abstract] OR "Viet Nam"[Title/Abstract] OR "Vietnam, Republic of"[Title/Abstract] OR "North Vietnam"[Title/Abstract] OR Srilanka[Title/Abstract])** | 12,06,180 |
| #1 AND #2 AND #3  (Year2004 – 2024) | **((("Osteoarthritis"[Mesh] OR "Osteoarthritis, Spine"[Mesh] OR "Osteoarthritis, Knee"[Mesh] OR "Osteoarthritis, Hip"[Mesh]) OR ("Osteoarthritis"[Title/Abstract] OR "Osteoarthrosis"[Title/Abstract] OR "osteoarthritides"[Title/Abstract] OR "arthritis degenerative"[Title/Abstract] OR "degenerative arthritis"[Title/Abstract] OR "arthros*"[Title/Abstract] OR "osteoarthrosis deformans"[Title/Abstract] OR "osteoarthritis of spine"[Title/Abstract] OR "spine osteoarthritis"[Title/Abstract] OR "spinal osteoarthritis"[Title/Abstract] OR "osteoarthritis spinal"[Title/Abstract] OR "osteoarthritis of the spine"[Title/Abstract] OR "lumbar osteoarthritis"[Title/Abstract] OR "osteoarthritis lumbar"[Title/Abstract] OR "knee osteoarthritis"[Title/Abstract] OR "osteoarthritis of knee"[Title/Abstract] OR "hip osteoarthritis"[Title/Abstract] OR "Coxarthrosis"[Title/Abstract] OR "Coxarthroses"[Title/Abstract] OR "osteoarthritis of the hip*"[Title/Abstract] OR "degenerative arthritis"[Title/Abstract] OR "degenerative joint disease"[Title/Abstract] OR "ankle osteoarthritis"[Title/Abstract] OR "Wear-and-tear arthritis"[Title/Abstract] OR "Joint degeneration"[Title/Abstract] OR "Cartilage degradation"[Title/Abstract] OR "Bone spurs"[Title/Abstract] OR "Osteophytes"[Title/Abstract] OR "Joint stiffness"[Title/Abstract] OR "Crepitus"[Title/Abstract] OR "Joint inflammation"[Title/Abstract] OR "Joint effusion"[Title/Abstract] OR "Hand osteoarthritis"[Title/Abstract] OR "Shoulder Osteoarthritis"[Title/Abstract] OR "Osteoarthritis Clinical Trials"[Title/Abstract])) AND (("Implementation Science"[Mesh] OR "Health Plan Implementation"[Mesh]) OR ("Implementation Science*"[Title/Abstract] OR "Health Plan Implementation*"[Title/Abstract] OR Implementation[Title/Abstract] OR implementing[Title/Abstract] OR implemented[Title/Abstract] OR operations[Title/Abstract] OR delivery[Title/Abstract] OR deliver[Title/Abstract] OR "translational science"[Title/Abstract] OR "translational research"[Title/Abstract] OR "Translational Medical Research"[Title/Abstract] OR "quality improvement"[Title/Abstract] OR "task shifting"[Title/Abstract] OR policy[Title/Abstract] OR monitoring[Title/Abstract] OR evaluation[Title/Abstract] OR "implementation process evaluation"[Title/Abstract] OR "impact evaluation"[Title/Abstract] OR "effectiveness, implementation"[Title/Abstract] OR "hybrid research"[Title/Abstract] OR "implementation studies"[Title/Abstract] OR "applied research"[Title/Abstract] OR "practice based research"[Title/Abstract] OR "operational research"[Title/Abstract] OR "applied implementation science"[Title/Abstract] OR "outcome evaluation"[Title/Abstract] OR "evidence based practice"[Title/Abstract] OR "policy implementation"[Title/Abstract] OR "stakeholder engagement"[Title/Abstract] OR "knowledge translation"[Title/Abstract] OR "contextual factors"[Title/Abstract] OR "barriers and facilitators"[Title/Abstract] OR "implementation strategies"[Title/Abstract] OR "implementation outcomes"[Title/Abstract] OR "real world implementation"[Title/Abstract] OR "implementation framework"[Title/Abstract] OR "Implementation fidelity"[Title/Abstract] OR "Implementation interventions"[Title/Abstract] OR "Implementation research methods"[Title/Abstract]))) AND (("Asia"[Mesh] OR Asia[Title/Abstract] OR "British Indian Ocean Territory"[Title/Abstract] OR Bangladesh[Title/Abstract] OR India[Title/Abstract] OR Bharat[Title/Abstract] OR "Republic of India"[Title/Abstract] OR "Khmer Republic"[Title/Abstract] OR Cambodia[Title/Abstract] OR Kampuchea[Title/Abstract] OR "Republic of China"[Title/Abstract] OR "Mainland China"[Title/Abstract] OR "Netherlands East Indies"[Title/Abstract] OR "East Indies"[Title/Abstract] OR "West Irian"[Title/Abstract] OR "New Guinea, Indonesian"[Title/Abstract] OR "New Guinea, West"[Title/Abstract] OR "Indonesian New Guinea"[Title/Abstract] OR "Irian Jaya"[Title/Abstract] OR "Federal Democratic Republic of Nepal"[Title/Abstract] OR "Islamic Republic of Pakistan"[Title/Abstract] OR Phillipines[Title/Abstract] OR Phillippines[Title/Abstract] OR Philipines[Title/Abstract] OR "Kingdom of Thailand"[Title/Abstract] OR Siam[Title/Abstract] OR "Viet Nam"[Title/Abstract] OR "Vietnam, Republic of"[Title/Abstract] OR "North Vietnam"[Title/Abstract] OR Srilanka[Title/Abstract])) Filters: from 2004 - 2024** | **351** |

**ProQuest – Total No of hits – 1764**

<https://www.proquest.com/search/2637028?accountid=173746>

| **S.no** | **Search terms** | **No of hits** |
| --- | --- | --- |
| S1 | Implementation research | [**860,122**](https://www.proquest.com/recentsearches.recentsearchtabview.recentsearchesgridview.scrolledrecentsearchlist.checkdbssearchlink_0:rerunsearch/9D1C47CE42FC4BA6PQ/None/$N?site=health&t:ac=RecentSearches) |
| S2 | Osteoarthritis | [**159,294**](https://www.proquest.com/recentsearches.recentsearchtabview.recentsearchesgridview.scrolledrecentsearchlist.checkdbssearchlink_0:rerunsearch/29E2F5EF037B440EPQ/None/$N?site=health&t:ac=RecentSearches) |
| S3 | Asia | [**496,461**](https://www.proquest.com/recentsearches.recentsearchtabview.recentsearchesgridview.scrolledrecentsearchlist.checkdbssearchlink_0:rerunsearch/D06DC5CD2AFB409APQ/None/$N?site=health&t:ac=RecentSearches) |
| S4 | [S1] AND [S2] AND [S3] (Year 2004-2024) | [**1,764**](https://www.proquest.com/recentsearches.recentsearchtabview.recentsearchesgridview.scrolledrecentsearchlist.checkdbssearchlink_0:rerunsearch/49B6C2439A924198PQ/None/$N?site=health&t:ac=RecentSearches) |

**CINAHL (EBSCO) – Total no of hits** - **711**

| **Serial no** | **Search terms** | **No of hits** |
| --- | --- | --- |
| S1 | (“Implementation Science*” OR “Health Plan Implementation*” OR Implementation OR implementing OR implemented OR operations OR delivery OR deliver OR “translational science” OR “translational research” OR “Translational Medical Research” OR “quality improvement” OR “task shifting” OR policy OR monitoring OR evaluation OR “implementation process evaluation” OR “impact evaluation” OR “effectiveness, implementation” OR “hybrid research” OR “applied implementation research” OR “implementation studies” OR “applied research” OR “practice based research” OR “operational research” OR “applied implementation science” OR “outcome evaluation” OR “evidence based practice” OR “policy implementation” OR “stakeholder engagement” OR “knowledge translation” OR “contextual factors” OR “barriers and facilitators” OR “implementation strategies” OR “implementation outcomes” OR “real world implementation” OR “implementation framework” OR “Implementation fidelity” OR “Implementation interventions” OR “Implementation research methods” OR “Translation of research into practice”) | 47,717 |
| S2 | (“Osteoarthritis" OR "Osteoarthrosis" OR "osteoarthritides" OR "arthritis degenerative" OR "degenerative arthritis" OR "arthros*" OR "osteoarthrosis deformans" OR "osteoarthritis of spine" OR "spine osteoarthritis" OR "spinal osteoarthritis" OR "osteoarthritis spinal" OR "osteoarthritis of the spine" OR "lumbar osteoarthritis" OR "osteoarthritis lumbar" OR "knee osteoarthritis" OR "osteoarthritis of knee" OR "hip osteoarthritis" OR "Coxarthrosis" OR "Coxarthroses" OR "osteoarthritis of the hip*" OR "degenerative arthritis" OR "degenerative joint disease" OR "ankle osteoarthritis" OR “Wear-and-tear arthritis” OR “Joint degeneration” OR “Cartilage degradation” OR “Bone spurs” OR “Osteophytes” OR “Joint stiffness” OR “Crepitus” OR “Joint inflammation” OR “Joint effusion” OR “Hand osteoarthritis” OR “Shoulder Osteoarthritis” OR “Physical Therapy for Osteoarthritis” OR “Inflammatory Mediators in Osteoarthritis” OR “Osteoarthritis Clinical Trials”) | 2,125 |
| S3 | (Asia OR "British Indian Ocean Territory" OR Bangladesh OR India OR Bharat OR "Republic of India" OR "Khmer Republic" OR Cambodia OR Kampuchea OR "Republic of China" OR "Mainland China" OR "Netherlands East Indies" OR "East Indies" OR "West Irian" OR "New Guinea, Indonesian" OR "New Guinea, West" OR "Indonesian New Guinea" OR "Irian Jaya" OR "Federal Democratic Republic of Nepal" OR "Islamic Republic of Pakistan" OR Phillipines OR Phillippines OR Philipines OR "Kingdom of Thailand" OR Siam OR "Viet Nam" OR "Vietnam, Republic of" OR "North Vietnam" OR Srilanka) | 161,757 |
| S1 AND S2 AND S3 (Year 2004-2024) | ( "implementation" OR "implementing" OR "Health Plan Implementation" OR "implemented" OR "operations" OR "delivery" OR "deliver" OR "implementation science" OR "translational science" OR "translational research" OR "translational medical research" OR "quality improvement" OR "task shifting" OR "policy" OR "monitoring" OR "evaluation" OR "process evaluation" OR "impact evaluation" OR "effectivenessimplementation" OR "hybrid research" ) AND ( “Osteoarthritis" OR "Osteoarthrosis" OR "osteoarthritides" OR "arthritis degenerative" OR "degenerative arthritis" OR "arthros*" OR "osteoarthrosis deformans" OR "osteoarthritis of spine" OR "spine osteoarthritis" OR "spinal osteoarthritis" OR "osteoarthritis spinal" OR "osteoarthritis of the spine" OR "lumbar osteoarthritis" OR "osteoarthritis lumbar" OR "knee osteoarthritis" OR "osteoarthritis of knee" OR "hip osteoarthritis" OR "Coxarthrosis" OR "Coxarthroses" OR "osteoarthritis of the hip*" OR "degenerative arthritis" OR "degenerative joint disease" OR "ankle osteoarthritis" ) AND ( Asia OR "British Indian Ocean Territory" OR Bangladesh OR India OR Bharat OR "Republic of India" OR "Khmer Republic" OR Cambodia OR Kampuchea OR "Republic of China" OR "Mainland China" OR "Netherlands East Indies" OR "East Indies" OR "West Irian" OR "New Guinea, Indonesian" OR "New Guinea, West" OR "Indonesian New Guinea" OR "Irian Jaya" OR "Federal Democratic Republic of Nepal" OR "Islamic Republic of Pakistan" OR Phillipines OR Phillippines OR Philipines OR "Kingdom of Thailand" OR Siam OR "Viet Nam" OR "Vietnam, Republic of" OR "North Vietnam" OR Srilanka ) | **711** |

**EMBASE – No of hits - 11**

| **Serial no** | **Search terms** | **No of hits** |
| --- | --- | --- |
| #1 | ('osteoarthritis'/exp OR 'arthritis, degenerative' OR 'arthritis, noninflammatory' OR 'arthrosis' OR 'degenerative arthritis' OR 'degenerative joint disease' OR 'noninflammatory arthritis' OR 'osteo-arthritis' OR 'osteo-arthrosis' OR 'osteoarthritis' OR 'osteoarthrosis' OR 'primary osteoarthritis' OR 'rheumatoid arthrosis' OR 'knee osteoarthritis'/exp OR 'arthrosis, knee' OR 'femorotibial arthrosis' OR 'gonarthrosis' OR 'knee arthrosis' OR 'knee joint arthrosis' OR 'knee joint osteoarthritis' OR 'knee osteo-arthritis' OR 'knee osteo-arthrosis' OR 'knee osteoarthritis' OR 'knee osteoarthrosis' OR 'osteoarthritis, knee' OR 'osteoarthrosis, knee' OR 'hip osteoarthritis'/exp OR 'arthrosis, hip' OR 'arthrosis, hip joint' OR 'cox arthrosis' OR 'coxartherosis' OR 'coxarthroses' OR 'coxarthrosis' OR 'hip arthrosis' OR 'hip joint arthrosis' OR 'hip osteo-arthritis' OR 'hip osteo-arthrosis' OR 'hip osteoarthritis' OR 'hip osteoarthrosis' OR 'malum coxae senilis' OR 'osteoarthritis, hip' OR 'sacroiliitis'/exp OR 'arthritis, iliosacral' OR 'iliosacral arthritis' OR 'osteoarthritis, sacroiliac' OR 'sacroileitis' OR 'sacroiliac arthritis' OR 'sacroiliac joint osteoarthritis' OR 'sacroiliac osteoarthritis' OR 'sacroiliitis' OR 'sacroilitis' OR 'experimental osteoarthritis'/exp OR 'experimental arthrosis' OR 'experimental osteo-arthritis' OR 'experimental osteoarthritis' OR 'experimentally induced osteoarthritis' OR 'osteoarthritis, experimental' OR 'osteoarthritis therapy'/exp) AND ('implementation science'/exp OR 'implementation research' OR 'implementation science' OR 'implementation scientific research' OR 'translational research'/exp OR 'translational bio-medical research' OR 'translational biomedical research' OR 'translational medical research' OR 'translational research' OR 'translational research, biomedical') AND [2004-2024]/py | 935 |
| #2 | **'Asia'** | 293,998 |
|  | #1 AND #2 (Year 2004-2024) | **11** |

**SCOPUS – Total no of hits - 133**

| **Serial no** | **Search terms** | **No of hits** |
| --- | --- | --- |
| #1 | ( INDEXTERMS ( "implementation science" ) OR INDEXTERMS ( "health plan implementation" ) ) OR ( TITLE-ABS ( "implementation science*" ) OR TITLE-ABS ( "health plan implementation*" ) OR TITLE-ABS ( implementation ) OR TITLE-ABS ( implementing ) OR TITLE-ABS ( implemented ) OR TITLE-ABS ( operations ) OR TITLE-ABS ( delivery ) OR TITLE-ABS ( deliver ) OR TITLE-ABS ( "translational science" ) OR TITLE-ABS ( "translational research" ) OR TITLE-ABS ( "translational medical research" ) OR TITLE-ABS ( "quality improvement" ) OR TITLE-ABS ( "task shifting" ) OR TITLE-ABS ( policy ) OR TITLE-ABS ( monitoring ) OR TITLE-ABS ( evaluation ) OR TITLE-ABS ( "implementation process evaluation" ) OR TITLE-ABS ( "impact evaluation" ) OR TITLE-ABS ( "effectiveness, implementation" ) OR TITLE-ABS ( "hybrid research" ) OR TITLE-ABS ( "implementation studies" ) OR TITLE-ABS ( "applied research" ) OR TITLE-ABS ( "practice based research" ) OR TITLE-ABS ( "operational research" ) OR TITLE-ABS ( "applied implementation science" ) OR TITLE-ABS ( "outcome evaluation" ) OR TITLE-ABS ( "evidence based practice" ) OR TITLE-ABS ( "policy implementation" ) OR TITLE-ABS ( "stakeholder engagement" ) OR TITLE-ABS ( "knowledge translation" ) OR TITLE-ABS ( "contextual factors" ) OR TITLE-ABS ( "barriers and facilitators" ) OR TITLE-ABS ( "implementation strategies" ) OR TITLE-ABS ( "implementation outcomes" ) OR TITLE-ABS ( "real world implementation" ) OR TITLE-ABS ( "implementation framework" ) OR TITLE-ABS ( "implementation fidelity" ) OR TITLE-ABS ( "implementation interventions" ) OR TITLE-ABS ( "implementation research methods" ) ) | [13,024,611](https://www.scopus.com/search/history/results.uri?origin=searchhistory&shid=6) |
| #2 | ( INDEXTERMS ( osteoarthritis ) OR INDEXTERMS ( "osteoarthritis, spine" ) OR INDEXTERMS ( "osteoarthritis, knee" ) OR INDEXTERMS ( "osteoarthritis, hip" ) ) OR ( TITLE-ABS ( osteoarthritis ) OR TITLE-ABS ( osteoarthrosis ) OR TITLE-ABS ( osteoarthritides ) OR TITLE-ABS ( "arthritis degenerative" ) OR TITLE-ABS ( "degenerative arthritis" ) OR TITLE-ABS ( arthros* ) OR TITLE-ABS ( "osteoarthrosis deformans" ) OR TITLE-ABS ( "osteoarthritis of spine" ) OR TITLE-ABS ( "spine osteoarthritis" ) OR TITLE-ABS ( "spinal osteoarthritis" ) OR TITLE-ABS ( "osteoarthritis spinal" ) OR TITLE-ABS ( "osteoarthritis of the spine" ) OR TITLE-ABS ( "lumbar osteoarthritis" ) OR TITLE-ABS ( "osteoarthritis lumbar" ) OR TITLE-ABS ( "knee osteoarthritis" ) OR TITLE-ABS ( "osteoarthritis of knee" ) OR TITLE-ABS ( "hip osteoarthritis" ) OR TITLE-ABS ( coxarthrosis ) OR TITLE-ABS ( coxarthroses ) OR TITLE-ABS ( "osteoarthritis of the hip*" ) OR TITLE-ABS ( "degenerative arthritis" ) OR TITLE-ABS ( "degenerative joint disease" ) OR TITLE-ABS ( "ankle osteoarthritis" ) OR TITLE-ABS ( "wear-and-tear arthritis" ) OR TITLE-ABS ( "joint degeneration" ) OR TITLE-ABS ( "cartilage degradation" ) OR TITLE-ABS ( "bone spurs" ) OR TITLE-ABS ( osteophytes ) OR TITLE-ABS ( "joint stiffness" ) OR TITLE-ABS ( crepitus ) OR TITLE-ABS ( "joint inflammation" ) OR TITLE-ABS ( "joint effusion" ) OR TITLE-ABS ( "hand osteoarthritis" ) OR TITLE-ABS ( "shoulder osteoarthritis" ) OR TITLE-ABS ( "osteoarthritis clinical trials" ) ) | [237,748](https://www.scopus.com/search/history/results.uri?origin=searchhistory&shid=7) |
| #3 | ( INDEXTERMS ( asia ) OR TITLE-ABS ( asia ) OR TITLE-ABS ( "british indian ocean territory" ) OR TITLE-ABS ( bangladesh ) OR TITLE-ABS ( india ) OR TITLE-ABS ( bharat ) OR TITLE-ABS ( "republic of india" ) OR TITLE-ABS ( "khmer republic" ) OR TITLE-ABS ( cambodia ) OR TITLE-ABS ( kampuchea ) OR TITLE-ABS ( "republic of china" ) OR TITLE-ABS ( "mainland china" ) OR TITLE-ABS ( "netherlands east indies" ) OR TITLE-ABS ( "east indies" ) OR TITLE-ABS ( "west irian" ) OR TITLE-ABS ( "new guinea, indonesian" ) OR TITLE-ABS ( "new guinea, west" ) OR TITLE-ABS ( "indonesian new guinea" ) OR TITLE-ABS ( "irian jaya" ) OR TITLE-ABS ( "federal democratic republic of nepal" ) OR TITLE-ABS ( "islamic republic of pakistan" ) OR TITLE-ABS ( phillipines ) OR TITLE-ABS ( phillippines ) OR TITLE-ABS ( philipines ) OR TITLE-ABS ( "kingdom of thailand" ) OR TITLE-ABS ( siam ) OR TITLE-ABS ( "viet nam" ) OR TITLE-ABS ( "vietnam, republic of" ) OR TITLE-ABS ( "north vietnam" ) OR TITLE-ABS ( srilanka ) ) | [1,018,616](https://www.scopus.com/search/history/results.uri?origin=searchhistory&shid=11) |
| #4 | ( ( INDEXTERMS ( "implementation science" ) OR INDEXTERMS ( "health plan implementation" ) ) OR ( TITLE-ABS ( "implementation science*" ) OR TITLE-ABS ( "health plan implementation*" ) OR TITLE-ABS ( implementation ) OR TITLE-ABS ( implementing ) OR TITLE-ABS ( implemented ) OR TITLE-ABS ( operations ) OR TITLE-ABS ( delivery ) OR TITLE-ABS ( deliver ) OR TITLE-ABS ( "translational science" ) OR TITLE-ABS ( "translational research" ) OR TITLE-ABS ( "translational medical research" ) OR TITLE-ABS ( "quality improvement" ) OR TITLE-ABS ( "task shifting" ) OR TITLE-ABS ( policy ) OR TITLE-ABS ( monitoring ) OR TITLE-ABS ( evaluation ) OR TITLE-ABS ( "implementation process evaluation" ) OR TITLE-ABS ( "impact evaluation" ) OR TITLE-ABS ( "effectiveness, implementation" ) OR TITLE-ABS ( "hybrid research" ) OR TITLE-ABS ( "implementation studies" ) OR TITLE-ABS ( "applied research" ) OR TITLE-ABS ( "practice based research" ) OR TITLE-ABS ( "operational research" ) OR TITLE-ABS ( "applied implementation science" ) OR TITLE-ABS ( "outcome evaluation" ) OR TITLE-ABS ( "evidence based practice" ) OR TITLE-ABS ( "policy implementation" ) OR TITLE-ABS ( "stakeholder engagement" ) OR TITLE-ABS ( "knowledge translation" ) OR TITLE-ABS ( "contextual factors" ) OR TITLE-ABS ( "barriers and facilitators" ) OR TITLE-ABS ( "implementation strategies" ) OR TITLE-ABS ( "implementation outcomes" ) OR TITLE-ABS ( "real world implementation" ) OR TITLE-ABS ( "implementation framework" ) OR TITLE-ABS ( "implementation fidelity" ) OR TITLE-ABS ( "implementation interventions" ) OR TITLE-ABS ( "implementation research methods" ) ) ) AND ( ( INDEXTERMS ( osteoarthritis ) OR INDEXTERMS ( "osteoarthritis, spine" ) OR INDEXTERMS ( "osteoarthritis, knee" ) OR INDEXTERMS ( "osteoarthritis, hip" ) ) OR ( TITLE-ABS ( osteoarthritis ) OR TITLE-ABS ( osteoarthrosis ) OR TITLE-ABS ( osteoarthritides ) OR TITLE-ABS ( "arthritis degenerative" ) OR TITLE-ABS ( "degenerative arthritis" ) OR TITLE-ABS ( arthros* ) OR TITLE-ABS ( "osteoarthrosis deformans" ) OR TITLE-ABS ( "osteoarthritis of spine" ) OR TITLE-ABS ( "spine osteoarthritis" ) OR TITLE-ABS ( "spinal osteoarthritis" ) OR TITLE-ABS ( "osteoarthritis spinal" ) OR TITLE-ABS ( "osteoarthritis of the spine" ) OR TITLE-ABS ( "lumbar osteoarthritis" ) OR TITLE-ABS ( "osteoarthritis lumbar" ) OR TITLE-ABS ( "knee osteoarthritis" ) OR TITLE-ABS ( "osteoarthritis of knee" ) OR TITLE-ABS ( "hip osteoarthritis" ) OR TITLE-ABS ( coxarthrosis ) OR TITLE-ABS ( coxarthroses ) OR TITLE-ABS ( "osteoarthritis of the hip*" ) OR TITLE-ABS ( "degenerative arthritis" ) OR TITLE-ABS ( "degenerative joint disease" ) OR TITLE-ABS ( "ankle osteoarthritis" ) OR TITLE-ABS ( "wear-and-tear arthritis" ) OR TITLE-ABS ( "joint degeneration" ) OR TITLE-ABS ( "cartilage degradation" ) OR TITLE-ABS ( "bone spurs" ) OR TITLE-ABS ( osteophytes ) OR TITLE-ABS ( "joint stiffness" ) OR TITLE-ABS ( crepitus ) OR TITLE-ABS ( "joint inflammation" ) OR TITLE-ABS ( "joint effusion" ) OR TITLE-ABS ( "hand osteoarthritis" ) OR TITLE-ABS ( "shoulder osteoarthritis" ) OR TITLE-ABS ( "osteoarthritis clinical trials" ) ) ) AND ( ( INDEXTERMS ( asia ) OR TITLE-ABS ( asia ) OR TITLE-ABS ( "british indian ocean territory" ) OR TITLE-ABS ( bangladesh ) OR TITLE-ABS ( india ) OR TITLE-ABS ( bharat ) OR TITLE-ABS ( "republic of india" ) OR TITLE-ABS ( "khmer republic" ) OR TITLE-ABS ( cambodia ) OR TITLE-ABS ( kampuchea ) OR TITLE-ABS ( "republic of china" ) OR TITLE-ABS ( "mainland china" ) OR TITLE-ABS ( "netherlands east indies" ) OR TITLE-ABS ( "east indies" ) OR TITLE-ABS ( "west irian" ) OR TITLE-ABS ( "new guinea, indonesian" ) OR TITLE-ABS ( "new guinea, west" ) OR TITLE-ABS ( "indonesian new guinea" ) OR TITLE-ABS ( "irian jaya" ) OR TITLE-ABS ( "federal democratic republic of nepal" ) OR TITLE-ABS ( "islamic republic of pakistan" ) OR TITLE-ABS ( phillipines ) OR TITLE-ABS ( phillippines ) OR TITLE-ABS ( philipines ) OR TITLE-ABS ( "kingdom of thailand" ) OR TITLE-ABS ( siam ) OR TITLE-ABS ( "viet nam" ) OR TITLE-ABS ( "vietnam, republic of" ) OR TITLE-ABS ( "north vietnam" ) OR TITLE-ABS ( srilanka ) ) ) AND PUBYEAR > 2003 AND PUBYEAR < 2025 | [**133**](https://www.scopus.com/search/history/results.uri?origin=searchhistory&shid=17) |

**WeB of Science** – Total no of hits - **216**

| **Serial no** | | **Search terms** | **No of hits** |
| --- | --- | --- | --- |
| #1 | (ALL="Implementation Science" OR ALL="Health Plan Implementation") OR ((TI="Implementation Science*" OR AB="Implementation Science*") OR (TI="Health Plan Implementation*" OR AB="Health Plan Implementation*") OR (TI=Implementation OR AB=Implementation) OR (TI=implementing OR AB=implementing) OR (TI=implemented OR AB=implemented) OR (TI=operations OR AB=operations) OR (TI=delivery OR AB=delivery) OR (TI=deliver OR AB=deliver) OR (TI="translational science" OR AB="translational science") OR (TI="translational research" OR AB="translational research") OR (TI="Translational Medical Research" OR AB="Translational Medical Research") OR (TI="quality improvement" OR AB="quality improvement") OR (TI="task shifting" OR AB="task shifting") OR (TI=policy OR AB=policy) OR (TI=monitoring OR AB=monitoring) OR (TI=evaluation OR AB=evaluation) OR (TI="implementation process evaluation" OR AB="implementation process evaluation") OR (TI="impact evaluation" OR AB="impact evaluation") OR (TI="effectiveness, implementation" OR AB="effectiveness, implementation") OR (TI="hybrid research" OR AB="hybrid research") OR (TI="implementation studies" OR AB="implementation studies") OR (TI="applied research" OR AB="applied research") OR (TI="practice based research" OR AB="practice based research") OR (TI="operational research" OR AB="operational research") OR (TI="applied implementation science" OR AB="applied implementation science") OR (TI="outcome evaluation" OR AB="outcome evaluation") OR (TI="evidence based practice" OR AB="evidence based practice") OR (TI="policy implementation" OR AB="policy implementation") OR (TI="stakeholder engagement" OR AB="stakeholder engagement") OR (TI="knowledge translation" OR AB="knowledge translation") OR (TI="contextual factors" OR AB="contextual factors") OR (TI="barriers and facilitators" OR AB="barriers and facilitators") OR (TI="implementation strategies" OR AB="implementation strategies") OR (TI="implementation outcomes" OR AB="implementation outcomes") OR (TI="real world implementation" OR AB="real world implementation") OR (TI="implementation framework" OR AB="implementation framework") OR (TI="Implementation fidelity" OR AB="Implementation fidelity") OR (TI="Implementation interventions" OR AB="Implementation interventions") OR (TI="Implementation research methods" OR AB="Implementation research methods")) | | 9,907,157 |
| #2 | (ALL=Osteoarthritis OR ALL="Osteoarthritis, Spine" OR ALL="Osteoarthritis, Knee" OR ALL="Osteoarthritis, Hip") OR ((TI=Osteoarthritis OR AB=Osteoarthritis) OR (TI=Osteoarthrosis OR AB=Osteoarthrosis) OR (TI=osteoarthritides OR AB=osteoarthritides) OR (TI="arthritis degenerative" OR AB="arthritis degenerative") OR (TI="degenerative arthritis" OR AB="degenerative arthritis") OR (TI=arthros* OR AB=arthros*) OR (TI="osteoarthrosis deformans" OR AB="osteoarthrosis deformans") OR (TI="osteoarthritis of spine" OR AB="osteoarthritis of spine") OR (TI="spine osteoarthritis" OR AB="spine osteoarthritis") OR (TI="spinal osteoarthritis" OR AB="spinal osteoarthritis") OR (TI="osteoarthritis spinal" OR AB="osteoarthritis spinal") OR (TI="osteoarthritis of the spine" OR AB="osteoarthritis of the spine") OR (TI="lumbar osteoarthritis" OR AB="lumbar osteoarthritis") OR (TI="osteoarthritis lumbar" OR AB="osteoarthritis lumbar") OR (TI="knee osteoarthritis" OR AB="knee osteoarthritis") OR (TI="osteoarthritis of knee" OR AB="osteoarthritis of knee") OR (TI="hip osteoarthritis" OR AB="hip osteoarthritis") OR (TI=Coxarthrosis OR AB=Coxarthrosis) OR (TI=Coxarthroses OR AB=Coxarthroses) OR (TI="osteoarthritis of the hip*" OR AB="osteoarthritis of the hip*") OR (TI="degenerative arthritis" OR AB="degenerative arthritis") OR (TI="degenerative joint disease" OR AB="degenerative joint disease") OR (TI="ankle osteoarthritis" OR AB="ankle osteoarthritis") OR (TI="Wear-and-tear arthritis" OR AB="Wear-and-tear arthritis") OR (TI="Joint degeneration" OR AB="Joint degeneration") OR (TI="Cartilage degradation" OR AB="Cartilage degradation") OR (TI="Bone spurs" OR AB="Bone spurs") OR (TI=Osteophytes OR AB=Osteophytes) OR (TI="Joint stiffness" OR AB="Joint stiffness") OR (TI=Crepitus OR AB=Crepitus) OR (TI="Joint inflammation" OR AB="Joint inflammation") OR (TI="Joint effusion" OR AB="Joint effusion") OR (TI="Hand osteoarthritis" OR AB="Hand osteoarthritis") OR (TI="Shoulder Osteoarthritis" OR AB="Shoulder Osteoarthritis") OR (TI="Osteoarthritis Clinical Trials" OR AB="Osteoarthritis Clinical Trials")) | | 196,591 |
| #3 | (ALL=Asia OR (TI=Asia OR AB=Asia) OR (TI="British Indian Ocean Territory" OR AB="British Indian Ocean Territory") OR (TI=Bangladesh OR AB=Bangladesh) OR (TI=India OR AB=India) OR (TI=Bharat OR AB=Bharat) OR (TI="Republic of India" OR AB="Republic of India") OR (TI="Khmer Republic" OR AB="Khmer Republic") OR (TI=Cambodia OR AB=Cambodia) OR (TI=Kampuchea OR AB=Kampuchea) OR (TI="Republic of China" OR AB="Republic of China") OR (TI="Mainland China" OR AB="Mainland China") OR (TI="Netherlands East Indies" OR AB="Netherlands East Indies") OR (TI="East Indies" OR AB="East Indies") OR (TI="West Irian" OR AB="West Irian") OR (TI="New Guinea, Indonesian" OR AB="New Guinea, Indonesian") OR (TI="New Guinea, West" OR AB="New Guinea, West") OR (TI="Indonesian New Guinea" OR AB="Indonesian New Guinea") OR (TI="Irian Jaya" OR AB="Irian Jaya") OR (TI="Federal Democratic Republic of Nepal" OR AB="Federal Democratic Republic of Nepal") OR (TI="Islamic Republic of Pakistan" OR AB="Islamic Republic of Pakistan") OR (TI=Phillipines OR AB=Phillipines) OR (TI=Phillippines OR AB=Phillippines) OR (TI=Philipines OR AB=Philipines) OR (TI="Kingdom of Thailand" OR AB="Kingdom of Thailand") OR (TI=Siam OR AB=Siam) OR (TI="Viet Nam" OR AB="Viet Nam") OR (TI="Vietnam, Republic of" OR AB="Vietnam, Republic of") OR (TI="North Vietnam" OR AB="North Vietnam") OR (TI=Srilanka OR AB=Srilanka)) | | 1,014,104 |
| #4 | (((ALL=Osteoarthritis OR ALL="Osteoarthritis, Spine" OR ALL="Osteoarthritis, Knee" OR ALL="Osteoarthritis, Hip") OR ((TI=Osteoarthritis OR AB=Osteoarthritis) OR (TI=Osteoarthrosis OR AB=Osteoarthrosis) OR (TI=osteoarthritides OR AB=osteoarthritides) OR (TI="arthritis degenerative" OR AB="arthritis degenerative") OR (TI="degenerative arthritis" OR AB="degenerative arthritis") OR (TI=arthros* OR AB=arthros*) OR (TI="osteoarthrosis deformans" OR AB="osteoarthrosis deformans") OR (TI="osteoarthritis of spine" OR AB="osteoarthritis of spine") OR (TI="spine osteoarthritis" OR AB="spine osteoarthritis") OR (TI="spinal osteoarthritis" OR AB="spinal osteoarthritis") OR (TI="osteoarthritis spinal" OR AB="osteoarthritis spinal") OR (TI="osteoarthritis of the spine" OR AB="osteoarthritis of the spine") OR (TI="lumbar osteoarthritis" OR AB="lumbar osteoarthritis") OR (TI="osteoarthritis lumbar" OR AB="osteoarthritis lumbar") OR (TI="knee osteoarthritis" OR AB="knee osteoarthritis") OR (TI="osteoarthritis of knee" OR AB="osteoarthritis of knee") OR (TI="hip osteoarthritis" OR AB="hip osteoarthritis") OR (TI=Coxarthrosis OR AB=Coxarthrosis) OR (TI=Coxarthroses OR AB=Coxarthroses) OR (TI="osteoarthritis of the hip*" OR AB="osteoarthritis of the hip*") OR (TI="degenerative arthritis" OR AB="degenerative arthritis") OR (TI="degenerative joint disease" OR AB="degenerative joint disease") OR (TI="ankle osteoarthritis" OR AB="ankle osteoarthritis") OR (TI="Wear-and-tear arthritis" OR AB="Wear-and-tear arthritis") OR (TI="Joint degeneration" OR AB="Joint degeneration") OR (TI="Cartilage degradation" OR AB="Cartilage degradation") OR (TI="Bone spurs" OR AB="Bone spurs") OR (TI=Osteophytes OR AB=Osteophytes) OR (TI="Joint stiffness" OR AB="Joint stiffness") OR (TI=Crepitus OR AB=Crepitus) OR (TI="Joint inflammation" OR AB="Joint inflammation") OR (TI="Joint effusion" OR AB="Joint effusion") OR (TI="Hand osteoarthritis" OR AB="Hand osteoarthritis") OR (TI="Shoulder Osteoarthritis" OR AB="Shoulder Osteoarthritis") OR (TI="Osteoarthritis Clinical Trials" OR AB="Osteoarthritis Clinical Trials"))) AND ((ALL="Implementation Science" OR ALL="Health Plan Implementation") OR ((TI="Implementation Science*" OR AB="Implementation Science*") OR (TI="Health Plan Implementation*" OR AB="Health Plan Implementation*") OR (TI=Implementation OR AB=Implementation) OR (TI=implementing OR AB=implementing) OR (TI=implemented OR AB=implemented) OR (TI=operations OR AB=operations) OR (TI=delivery OR AB=delivery) OR (TI=deliver OR AB=deliver) OR (TI="translational science" OR AB="translational science") OR (TI="translational research" OR AB="translational research") OR (TI="Translational Medical Research" OR AB="Translational Medical Research") OR (TI="quality improvement" OR AB="quality improvement") OR (TI="task shifting" OR AB="task shifting") OR (TI=policy OR AB=policy) OR (TI=monitoring OR AB=monitoring) OR (TI=evaluation OR AB=evaluation) OR (TI="implementation process evaluation" OR AB="implementation process evaluation") OR (TI="impact evaluation" OR AB="impact evaluation") OR (TI="effectiveness, implementation" OR AB="effectiveness, implementation") OR (TI="hybrid research" OR AB="hybrid research") OR (TI="implementation studies" OR AB="implementation studies") OR (TI="applied research" OR AB="applied research") OR (TI="practice based research" OR AB="practice based research") OR (TI="operational research" OR AB="operational research") OR (TI="applied implementation science" OR AB="applied implementation science") OR (TI="outcome evaluation" OR AB="outcome evaluation") OR (TI="evidence based practice" OR AB="evidence based practice") OR (TI="policy implementation" OR AB="policy implementation") OR (TI="stakeholder engagement" OR AB="stakeholder engagement") OR (TI="knowledge translation" OR AB="knowledge translation") OR (TI="contextual factors" OR AB="contextual factors") OR (TI="barriers and facilitators" OR AB="barriers and facilitators") OR (TI="implementation strategies" OR AB="implementation strategies") OR (TI="implementation outcomes" OR AB="implementation outcomes") OR (TI="real world implementation" OR AB="real world implementation") OR (TI="implementation framework" OR AB="implementation framework") OR (TI="Implementation fidelity" OR AB="Implementation fidelity") OR (TI="Implementation interventions" OR AB="Implementation interventions") OR (TI="Implementation research methods" OR AB="Implementation research methods")))) AND ((ALL=Asia OR (TI=Asia OR AB=Asia) OR (TI="British Indian Ocean Territory" OR AB="British Indian Ocean Territory") OR (TI=Bangladesh OR AB=Bangladesh) OR (TI=India OR AB=India) OR (TI=Bharat OR AB=Bharat) OR (TI="Republic of India" OR AB="Republic of India") OR (TI="Khmer Republic" OR AB="Khmer Republic") OR (TI=Cambodia OR AB=Cambodia) OR (TI=Kampuchea OR AB=Kampuchea) OR (TI="Republic of China" OR AB="Republic of China") OR (TI="Mainland China" OR AB="Mainland China") OR (TI="Netherlands East Indies" OR AB="Netherlands East Indies") OR (TI="East Indies" OR AB="East Indies") OR (TI="West Irian" OR AB="West Irian") OR (TI="New Guinea, Indonesian" OR AB="New Guinea, Indonesian") OR (TI="New Guinea, West" OR AB="New Guinea, West") OR (TI="Indonesian New Guinea" OR AB="Indonesian New Guinea") OR (TI="Irian Jaya" OR AB="Irian Jaya") OR (TI="Federal Democratic Republic of Nepal" OR AB="Federal Democratic Republic of Nepal") OR (TI="Islamic Republic of Pakistan" OR AB="Islamic Republic of Pakistan") OR (TI=Phillipines OR AB=Phillipines) OR (TI=Phillippines OR AB=Phillippines) OR (TI=Philipines OR AB=Philipines) OR (TI="Kingdom of Thailand" OR AB="Kingdom of Thailand") OR (TI=Siam OR AB=Siam) OR (TI="Viet Nam" OR AB="Viet Nam") OR (TI="Vietnam, Republic of" OR AB="Vietnam, Republic of") OR (TI="North Vietnam" OR AB="North Vietnam") OR (TI=Srilanka OR AB=Srilanka))) | | **216** |

**Science Direct -897**
