# Supplementary material for: Implementation research on osteoarthritis in Asia: a systematic review
Source: Front Public Health. 2026 Feb 10;14:1693849. doi: 10.3389/fpubh.2026.1693849 (PMC12931281; doi:10.3389/fpubh.2026.1693849)
Supplement: Supplementary file 2 [file Table_2.DOCX]

**Supplementary Table S2:** StaRI checklist (Reporting quality assessment)

| **Studies** | **Implementation score** | **Reporting completeness*** | **Intervention score** | **Reporting completeness*** |
| --- | --- | --- | --- | --- |
| Goh et al. (2014) | 21 | High | 20 | High |
| Opava et al. (2023) | 21 | High | 21 | High |
| Thapa et al. (2024) | 18 | Moderate | 21 | High |
| Aree-Ue et al. (2022) | 18 | Moderate | 19 | Moderate |
| Batra et al. (2010) | 15 | Moderate | 15 | Moderate |
| Lee et al. (2016) | 13 | Low | 20 | High |
| Yul et al. (2012) | 18 | Moderate | 19 | Moderate |

***Reporting completeness (based on total score out of 27):**

- **High**: ≥ 20 items
- **Moderate**: 15–19 items
- **Low**: < 15 items

**#StaRI checklist:** https://www.equator-network.org/reporting-guidelines/stari-statement/
