# Supplementary material for: Implementation research on osteoarthritis in Asia: a systematic review
Source: Front Public Health. 2026 Feb 10;14:1693849. doi: 10.3389/fpubh.2026.1693849 (PMC12931281; doi:10.3389/fpubh.2026.1693849)
Supplement: Supplementary file 3 [file Table_3.DOCX]

**Identification of studies via databases and registers**

**Screening**

**Identification**

#

**Records identified from:**

PubMed (n = 355)

Embase (n = 11)

EBSCO (n = 643)

Web of Science (n = 208)

Scopus (n = 134)

ScienceDirect (n = 914)

ProQuest (n = 100)

Google scholar (n = 0)

Shodhganga (n = 0)

Records removed before screening:

Duplicate records removed

(n = 214)

Records screened for title and abstract (n = 2151)

Records excluded (n = 2131)

Reports not retrieved (n = 0)

Reports sought for retrieval (n = 20)

Reports excluded: (n= 13)

- No IR component (n = 8)
- Wrong population (n = 5)

Reports assessed for eligibility (n = 20)

Studies included in review (n = 7)

**Included**

**Figure 1:** Flow chart depicting the systematic review process
